# Prognostic relevance of mitral and tricuspid regurgitation after transcatheter aortic valve implantation: Impact of follow-up time point for decision-making

**DOI:** 10.3389/fcvm.2023.990373

**Published:** 2023-02-16

**Authors:** Laura Bäz, Sven Möbius-Winkler, Mahmoud Diab, Thomas Kräplin, Julian G. Westphal, Karim Ibrahim, P. Christian Schulze, Marcus Franz

**Affiliations:** ^1^Department of Internal Medicine I, University Hospital Jena, Jena, Germany; ^2^Research Program “Else Kröner-Forschungskolleg AntiAge”, Jena University Hospital, Jena, Germany; ^3^Department of Cardiothoracic Surgery, University Hospital Jena, Jena, Germany; ^4^University Hospital Jena, Jena, Germany; ^5^Department of Internal Medicine I, Klinikum Chemnitz, Chemnitz, Germany

**Keywords:** transcatheter aortic valve implantation, mitral regugitation, tricuspid regurgitation, prognosis, treatment

## Abstract

**Background:**

In patients with aortic stenosis treated by transcatheter aortic valve implantation (TAVI), mitral and tricuspid regurgitation (MR and TR) at baseline and after TAVI are likely to be of prognostic relevance, and questions such as whether and when treatment further improves prognosis in these patients arise.

**Aims:**

Against that background, the purpose of this study was to analyze a variety of clinical characteristics including MR and TR with respect to their potential value as predictors of 2-year mortality after TAVI.

**Methods:**

A cohort of 445 typical TAVI patients was available for the study and clinical characteristics were evaluated baseline, 6 to 8 weeks as well as 6 months after TAVI.

**Results:**

In 39% of the patients relevant (moderate or severe) MR and in 32% of the patients relevant (moderate or severe) TR could be detected at baseline. The rates were 27% for MR (*p* = 0.001, compared to baseline) and 35% for TR (*p* = n.s., compared to baseline) at the 6- to 8-week follow-up. After 6 months, relevant MR was observable in 28% (*p* = 0.036, compared to baseline) and relevant TR in 34% (*p* = n.s., compared to baseline) of the patients. As predictors of 2-year mortality, a multivariate analysis identified the following parameters for the different time points: sex, age, AS entity, atrial fibrillation, renal function, relevant TR, systolic pulmonary artery pressure (PAPsys), and 6-min walk distance at baseline; clinical frailty scale and PAPsys 6–8 weeks after TAVI and BNP and relevant MR 6 months after TAVI. There was a significantly worse 2-year survival in patients with relevant TR at baseline (68.4% vs. 82.6%, *p* < 0.001; whole population, *n* = 445) and in patients with relevant MR at 6 months (87.9% vs. 95.2%, *p* = 0.042; landmark analysis: *n* = 235).

**Conclusion:**

This real-life study demonstrated the prognostic relevance of repeated evaluation of MR and TR before and after TAVI. Choosing the right time point for treatment is a remaining clinical challenge, which should be further addressed in randomized trials.

## Introduction

Transcatheter aortic valve implantation (TAVI) is nowadays considered the state-of-the-art treatment for elderly patients suffering from severe aortic stenosis (AS) in the symptomatic stage. In addition, low risk patients not suitable for surgical aortic valve replacement (SAVR) could be shown to be effectively treated by TAVI (1–8). Typical patients with TAVI suffer from a variety of comorbidities, e.g., atrial fibrillation, renal dysfunction, diabetes mellitus, coronary artery disease (CAD), heart failure, or chronic obstructive pulmonary disease, which all could be proven to be independent predictors of long-term outcome and mortality and have therefore been integrated into several risk prediction models and scores developed and validated recently (9–14). Unfortunately, most of these risk factors and comorbidities cannot be causally treated by a concomitantly or consecutively performed dedicated intervention further improving long-term prognosis. The coexistence of AS and mitral regurgitation (MR) at baseline is frequent and likely to be of prognostic relevance after successful TAVI but could not convincingly be proven to be an independent predictor of long-term mortality in most studies until now (15–18). Furthermore, in a large nationwide database-derived cohort study in France including 42.866 patients, baseline MR was not independently associated with all-cause or cardiovascular mortality in patients with TAVI (19). Nevertheless, there is one meta-analysis, in which relevant baseline MR was associated with higher mortality rates both, at 30 days as well as 1 year after TAVI (20).

In addition to MR, also relevant baseline tricuspid regurgitation (TR) has been reported to occur in patients before TAVI but is less frequently observable and often associated with group 2 pulmonary hypertension (PH) or right ventricular (RV) remodeling and dysfunction (21). In case of significant (moderate or severe) TR combined with decreased right ventricular function before TAVI, the prognosis could be demonstrated to be impaired with increased long-term mortality rates (22, 23). In contrast, in the aforementioned large French cohort study, significant baseline TR could not be proven to be an independent mortality risk predictor (19).

The missing link between baseline MR might be because the majority of MR is functional and probably, at least in part, caused by AS (24, 25). Thus, MR severity is likely to be extremely dynamic after TAVI in response to a complex process of reverse remodeling secondary to decreased afterload of the left ventricle (26, 27). This might, to a lesser extent, also be true for relevant baseline TR, which may also decrease after TAVI (28, 29). However, in contrast to MR, the situation seems to be much more complex and less conceivable since TR severity and reversibility are mainly dependent on the extent of group 2 PH with or without precapillary component as well as RV function (21, 30, 31). Taken together, the question of whether and when patients might benefit from additional MR or TR treatment in addition to TAVI is of certain interest and remains challenging in daily clinical practice.

### Aim of the study

The current study aimed to identify predictors of long-term mortality assessed before and after TAVI with a special focus on MR and TR in a prospective real-world single-center registry study adhering to a structured clinical, laboratory, and echocardiographic follow-up algorithm.

## Methods

### Study population and data collection

In this prospective real-world single-center registry study, a total of 445 patients with severe symptomatic degenerative AS undergoing transcatheter aortic valve implantation (TAVI) according to current guidelines and after discussion with the heart team (8, 32) were included. All patients gave written informed consent to participate in the Jenaer Aortenklappenregister (JAKR) which was established in 2016 for consecutive inclusion of all patients undergoing TAVI at the University Hospital Jena. The study was approved by the local ethics committee (registration number: 4815–06/16). The only cases that have been excluded from this analysis were patients suffering from pure aortic regurgitation and undergoing TAVI due to inoperability (<10 cases within the study period).

A large set of clinical, laboratory, functional, and imaging parameters was prospectively assessed according to local standard operating procedures and the JAKR study protocol (33). In addition, the clinical frailty scale (CFS) and visual analogue scale (VAS) were assessed at each time point (34, 35). Survival of patients was recorded at 30 days, 1 year, and 2 years after TAVI. All investigations were performed in strict adherence to good clinical practice guidelines and the principles of the current version of the Declaration of Helsinki.

Transthoracic echocardiography (TTE) was performed at baseline and at all follow-up time points after TAVI according to a standardized protocol adhering to the recommendations of the American Society of Echocardiography and current guidelines (8, 36, 37). Analyses were done by experienced analysts who independently performed echocardiography for at least 2 years at the time point of assessment (*κ* > 0.81 for inter-observer variability; *κ* > 0.9 for intra-observer variability).

### Statistical analysis

Statistical analyses were performed using IBM SPSS statistical software, version 28.0 (IBM SPSS Statistics for Windows. Armonk, NY, USA). Data are expressed as mean ± standard deviation or median and interquartile range, as appropriate, in case of continuous variables. Categorical variables are given as percentages.

The Wilcoxon test was used to assess significant differences in clinical, laboratory, and echocardiographic parameters before TAVI (baseline, *n* = 445) compared to/between the follow-up time points *6 to 8 weeks* (*n* = 334, 75.1%) and *6 months* (*n* = 235, 52.8%) after TAVI (dependent variables).

The Mann–Whitney U-test was used to test for significant differences in clinical, laboratory, and echocardiographic parameters between long-term survivors and non-survivors with respect to 2-year mortality at the different time points (*baseline*, *6 to 8 weeks*, and *6 months*). The parameters assessed at baseline were divided into parameters unlikely to change after TAVI (designated as *static*) and parameters showing dynamics after TAVI (designated as *dynamic*). Thus, there were four clusters of parameters: *baseline static*, *baseline dynamic*, *6- to 8-week follow-up,* and *6-month follow-up*. Those parameters showing a value of p of <0.1 between long-term survivors and non-survivors after 2 years in Mann–Whitney U-test (Supplementary Table 1) were included in a multivariate analysis for each cluster to identify independent risk predictors for long-term (2-year) mortality. Therefore, multivariate regression analyses were performed by using a binary logistic model (backward elimination method: Wald) for each of the four clusters. Long-term mortality was defined as the dependent variable. Thereafter, multistep backward elimination (removal threshold *p* > 0.10) of independent variables was carried out. A value of p of <0.05 was considered statistically significant.

A Kaplan–Meier survival analysis including a log-rank test was performed to verify differences between patients with none or mild versus moderate or severe MR (at 6-month follow-up) and TR (at baseline) as well as relevant paravalvular leakage (PVL, at 6-month follow-up) with respect to long-term (2-year) mortality (selection of parameters according to the results of multivariate analysis and study focus).

## Results

### Description of the study cohort

The study cohort represented a typical TAVI patient collective (mean age: 78.7 ± 7.3 years, 52% female; mean STS score 5 ± 3.9%). All patients were successfully treated by TAVI *via* the transfemoral access route using balloon-expandable (60.1%) or self-expanding (39.9%) prostheses. With respect to AS subtype, 74% were classified as high-, 16% as low-, and 10% as paradoxical low-flow low-gradient AS. In 39% of the patients relevant (moderate or severe) MR (97.3% secondary/functional) and in 32% of the patients relevant (moderate or severe) TR (95.8% secondary/functional) could be detected at baseline. Table 1 summarizes baseline characteristics and Table 2 summarizes outcome measures of the 445 patients included in the study (Tables 1, 2).

**Table 1:** Baseline characteristics of the study cohort.

Parameter	Study cohort (*n* = 445)
Age (years; mean ± SD)	78.7 ± 7.3
Height (cm; mean ± SD)	167 ± 10
Weight (kg; mean ± SD)	77 ± 17
Female (%)	52
STS (%; mean ± SD)	5.0 ± 3.9
AS subtype (%)	
HGAS	74
LGAS	16
PLFLGAS	10
Staging of extra-valvular cardiac damage 0–4 (%) (24)	
0	2.9
1	12.4
2	49.2
3	22.3
4	13.1
Comorbidities	
CAD (%)	61.4
PAD (%)	12.4
Diabetes (%)	42.2
COPD (%)	26.0
Afib (%)	50.7
PM (%)	14.5
CKD (GFR in mL/min; mean ± SD)	53.2 ± 22.6
PH (%)	27.1
LC (%)	2.9
TAVI prosthesis	
Balloon-expandable TAVI prosthesis (%)	60.1
Self-expanding TAVI prosthesis (%)	39.9

STS score, Society of Thoracic Surgeons score; AS, Aortic stenosis; HGAS, High-gradient aortic stenosis; LGAS, Low-gradient aortic stenosis; PLFLGAS, Paradoxical low-flow low-gradient aortic stenosis; stage, stages of extra-valvular cardiac damage; CAD, Coronary artery disease; PAD, Peripheral artery disease; COPD, Chronic obstructive pulmonary disease; Afib, Atrial fibrillation; PM, Pacemaker; CKD, Chronic kidney disease; GFR, Glomerular filtration rate; PH, Pulmonary hypertension; LC, Liver cirrhosis.

**Table 2:** Outcome measures of the study cohort.

Parameter	Baseline	*p* value	6 to 8 weeks follow up	*p* value	6 months follow up	*p* value
		(6 to 8 weeks compared to baseline)		(6 months compared to 6 to 8 weeks)		(6 months compared to baseline)
NYHA > II (%)	72.8	<0.001	12.4	0.02	9.6	<0.001
BNP (pg/ml; mean ± SD)	841 ± 1,637	<0.001	374 ± 576	0.06	287 ± 354	<0.001
SMWD (meters; mean ± SD)	213 ± 159	<0.001	288 ± 132	0.36	299 ± 118	<0.001
LVEF (%; mean ± SD)	58 ± 15	<0.001	62 ± 13	1	63 ± 12	<0.001
Impaired RV-function (%)	16	<0.001	3	0	6	0
PAPsys (mmHg; mean ± SD)	41 ± 13	<0.001	35 ± 12	0.56	35 ± 12	<0.001
MR ≥ II (%)	39	0	27	0.17	28	0.04
TR ≥ II (%)	32	0.26	35	0.62	34	0.27
CFS (points; mean ± SD)	4.0 ± 1.1	<0.001	3.5 ± 1.3	0.7	3.5 ± 1.1	0.45
VAS – self - rated health (points; mean ± SD)	57 ± 18	<0.001	63 ± 18	0.45	64 ± 18	0.7

NYHA, New York Heart Association; BNP, Brain natriuretic peptide; SMWD, 6-min walk distance; LVEF, Left ventricular ejection fraction; RV function, right ventricular function; PAPsys, Systolic pulmonary artery pressure; MR, Mitral regurgitation; TR, Tricuspid regurgitation; CFS, Clinical frailty scale; VAS, Visual analogue scale.

### Outcome after TAVI

**Figure 1:**
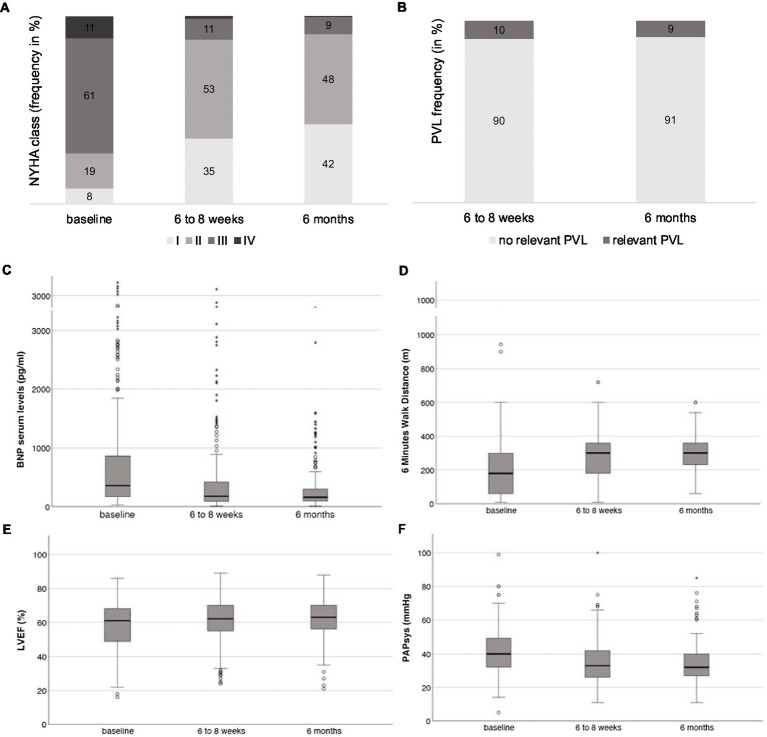
(**A**) Distribution of NYHA functional classes I to IV at baseline, the 6- to 8-week, and the 6-month follow-up. (**B**) Frequency of relevant PVL at the 6- to 8-week and the 6-month follow-up. Development of (**C**) BNP serum levels, (**D**) SMWD, (**E**) LVEF, and (**F**) PAPsys at baseline, the 6- to 8-week, and the 6-month follow-up.

The TAVI procedure could be performed successfully in all patients consecutively included in the study between August 2016 and July 2019. Mortality was 3.6% after 30 days, 16.4% after 1 year, and 22.6% after 2 years. At the first follow-up 6 to 8 weeks after TAVI, there was a significant improvement in NYHA functional class (Figure 1A), brain natriuretic peptide (BNP) serum levels, 6-min walk distance (SMWD), left ventricular ejection fraction (LVEF), and systolic pulmonary artery pressure (PAPsys) determined by TTE as compared to baseline (*p* < 0.01 for all parameters, Figures 1C–F). There was a further improvement in NYHA functional class (*p* = 0.018) and, at least in trend, of BNP serum levels (*p* = 0.058), when comparing the 6-month and the 6- to 8-week follow-up. The remaining parameters presented stability with significant improvements compared to the baseline (p < 0.01). The percentage of relevant paravalvular leakage (PVL, resulting in moderate or severe aortic regurgitation) was 10% after 6 to 8 weeks and 9% after 6 months (*p* = 0.251) (Figure 1B).

### Dynamics of MR and TR following TAVI

**Figure 2:**
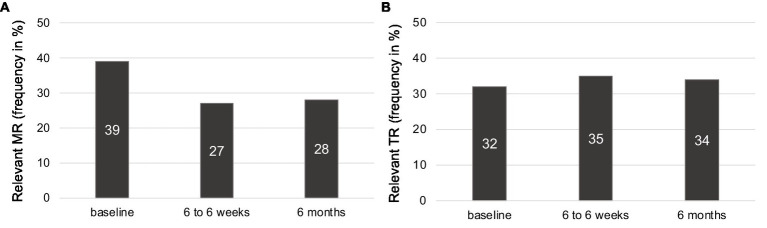
Frequency of relevant MR (**A**) and TR (**B**) at baseline, the 6- to 8-week, and the 6-month follow-up.

After 6 to 8 weeks, the frequency of relevant MR was 27% (compared to 39% at baseline, *p* = 0.001) and of relevant TR 35% (compared to 32% at baseline, *p* = 0.259). For relevant MR, the frequency after 6 months was 28% (*p* = 0.036, compared to baseline) and 34% for relevant TR (*p* = 0.267, compared to baseline) (Figures 2A,B).

### Risk predictors of long-term mortality.

The following predictors of 2-year mortality could be identified by multivariate analysis: age, sex, AS subtype other than high-gradient, atrial fibrillation, and renal function in the cluster of *baseline_static*; relevant TR, PAPsys, and SMWD in the cluster of *baseline_dynamic*; CFS and PAPsys in the cluster of *6- to 8-week follow-up;* and BNP and MR in the *cluster 6 months follow-up*. Detailed data (Exp(B) = odds ratios (OR), 95% confidence intervals (95% CI), and value of ps) are given in Table 3.

**Table 3:** Risk predictors of long-term (2-year) mortality.

Parameters	Exp (*B*) = odds ratio (OR)	95% confidence interval (95% CI)	*p* value
*baseline_static*			
Age	1.032	0.994–1.070	0.098
Female sex	0.631	0.381–1.043	0.073
AS subtype, other than HGAS	1.615	0.955–2.730	0.074
Afib	2.505	1.509–4.157	**<0.001**
Renal function (GFR)	0.985	0.974–0.996	**0.009**
*baseline_dynamic*			
SMWD	0.996	0.992–1.000	0.053
PAPsys	1.032	0.995–1.071	0.093
TR ≥ II	2.179	1.105–4.299	**0.025**
*6 to 8 weeks follow-up*			
CFS	1.841	1.117–3.037	**0.017**
PAPsys	1.067	1.017–1.119	**0.008**
*6 months follow-up*			
BNP	1.001	1.000–1.003	0.081
MR ≥ II	3.192	0.971–10.487	0.056

AS, Aortic stenosis; HGAS, High-gradient aortic stenosis; Afib, Atrial fibrillation; GFR, Glomerular filtration rate; SMWD, 6-min walk distance; PAPsys, Systolic pulmonary artery pressure; TR, Tricuspid regurgitation; CFS, Clinical frailty scale; BNP, Brain natriuretic peptide; MR, Mitral regurgitation. Bold values indicate statistical significant *p* values.

### Survival analysis using Kaplan–Meier estimate

**Figure 3:**
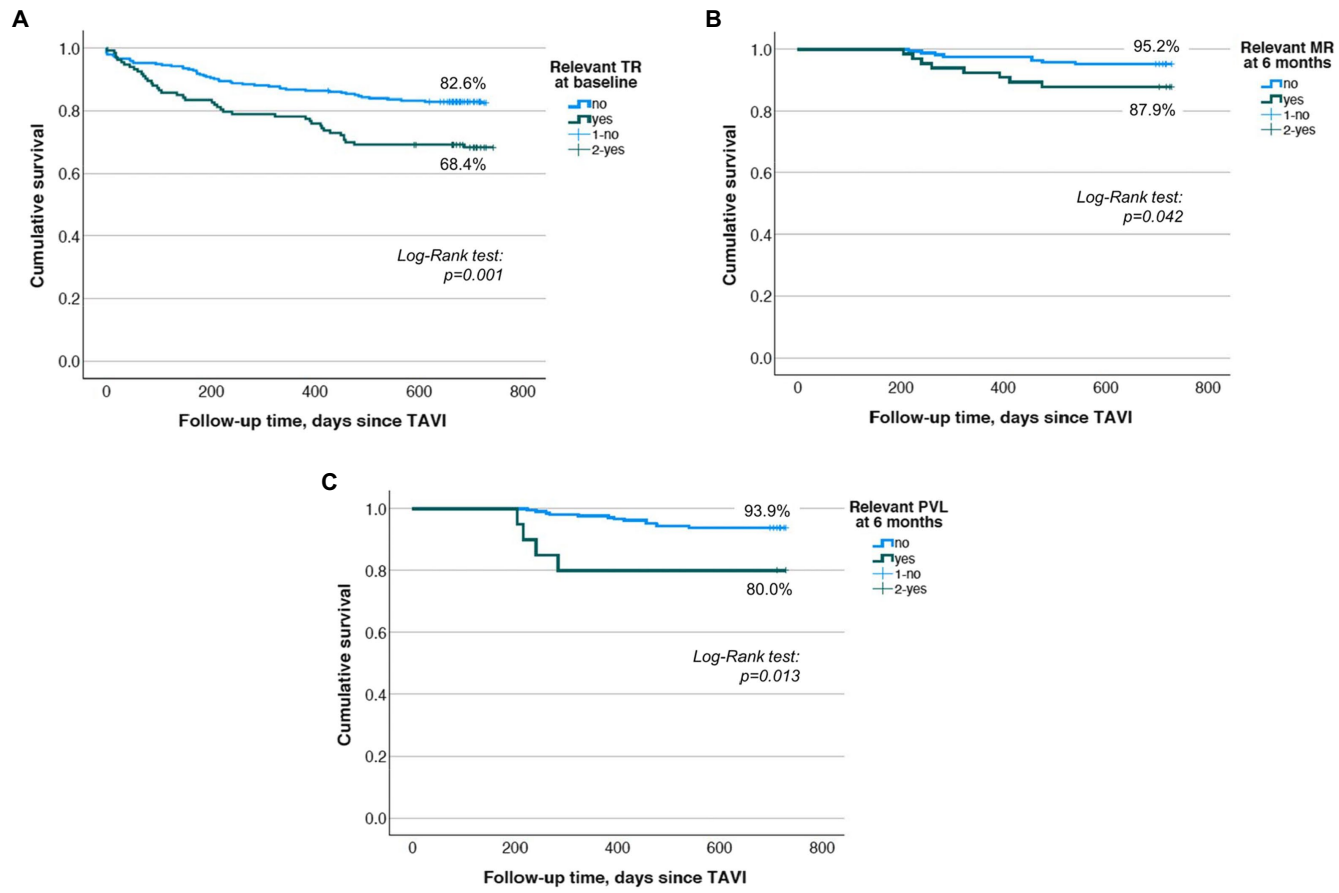
(**A**) Kaplan–Meier survival analysis for relevant TR at baseline displaying a significant survival benefit after 2 years for the group not showing relevant TR at baseline. (**B**) Kaplan–Meier survival analysis for relevant MR at the 6-month follow-up displaying a significant survival benefit after 2 years for the group not showing relevant MR 6 months after TAVI. (**C**) Kaplan–Meier survival analysis for relevant PVL at the 6-month follow-up displaying a significant survival benefit after 2 years for the group not showing relevant PVL 6 months after TAVI.

There was a significantly worse 2-year survival in patients suffering from relevant TR at baseline (68.4% vs. 82.6%, *p* < 0.001; whole population, *n* = 445; Figure 3A) and in those with relevant MR at 6 months (87.9% vs. 95.2%, *p* = 0.042; landmark analysis: *n* = 235; Figure 3B) as displayed by the Kaplan–Meier survival analysis. Survival rates were significantly lower in patients showing relevant PVL at 6 months (80% vs. 93.9%, *p* = 0.013; landmark analysis: *n* = 235; Figure 3C). However, PVL could not be shown to serve as an independent mortality predictor.

## Discussion

In this prospective real-world registry study including 445 moderate- to high-risk TAVI patients, a persisting improvement of concomitant MR but not TR was observable already after 6 to 8 weeks with no further dynamics after 6 months. Interestingly, a relevant TR but not MR before TAVI (baseline) as well as a relevant MR but not TR at the 6-month follow-up were independent predictors of 2-year mortality. The latter interrelation could not be shown for the short-term, 6- to 8-week follow-up.

The current analysis is based on a large single-center registry with observed mortality rates of 16.4 and 22.6% after 1 and 2 years, respectively. These rates are equal to outcomes of large randomized clinical trials and real-world registry studies including intermediate or high risk patients. Thus, the PARTNER 2 trial (intermediate risk patients) reported mortality rates of 12.3% after 1 year and 16.7% after 2 years, and the CoreValve US Trial observed 2-year mortality of 22.2% (3, 38). In a large US registry including more than 12,000 patients, 1-year mortality after TAVI was 23.7% (39).

While baseline MR was not independently associated with increased long-term mortality in our study, there are conflicting results reported in the literature. Thus, a variety of studies could observe relevant MR as an independent predictor of long-term mortality at 1 year and beyond (25, 40), whereas others could not report on such interrelations (16, 19, 41). The phenomenon that MR in patients with severe AS has the potential to improve, to present unchanged, or even to worsen after TAVI has been widely described recently (29, 42) and has been shown again in this analysis. However, the majority of studies addressing the prognostic relevance of significant MR in patients with TAVI are solely focused on the baseline conditions leading to very limited knowledge about the prognostic impact of post-procedural MR dynamics. Nevertheless, in a Swedish registry study including 1,712 patients, MR improvement after TAVI was associated with increased survival rates at long-term follow-up compared to patients showing persisting significant or worsened MR (43).

Interestingly, in contrast to baseline and the 6- to 8-week follow-up, relevant MR at 6 months was associated with increased 2-year mortality in our study. This goes in line with a recent analysis of Ben-Assa and co-workers published in 2020, which could show post- but not pre-procedural relevant MR to be associated with increased long-term mortality after TAVI in a cohort of 486 patients who underwent TAVI between 2009 and 2014. In congruence with our findings, the hemodynamic impact of relevant MR in this study could be shown to be manifest not until 6 months post-TAVI (42).

Moreover, a large multicenter analysis (AMTRAC registry) published in 2021 could show that mortality in patients presenting with persistent relevant MR 30 days after TAVI was higher compared to patients not showing relevant MR before TAVI. The fact that we could not show this effect at the 6- to 8-week follow-up but at 6 months after TAVI might be due to the lower subject number in our single-center study and differences in statistical approaches, e.g., the reference group, which was patients without relevant MR before TAVI and not patients without relevant MR at 6 to 8 weeks as in our study. In addition, 4-year mortality was compared, while our analysis focused on 2-year mortality (44).

Whereas the study size is comparable to our current analysis including 445 patients, one has to notice that patients investigated by us underwent TAVI between 2016 and 2019 with new-generation prostheses; wherefore, comparability might be limited. This is of special importance since there is clear evidence that the technical evolution of TAVI devices is associated with lower complication rates and better outcomes (45).

For TR, data are contradictory with both, studies speaking for or against improvement after TAVI. This is probably due to the more complex etiology of TR being dependent on the extent of group 2 pulmonary hypertension (PH) with or without a precapillary component and right ventricular function. Thus, our observation of stable TRs after TAVI without improvement at follow-up is not surprising since more than one-third (35.4%) of patients in this study exhibited extended stages (3 or 4) of extra-valvular cardiac damage according to the staging classification published in 2017 for the PARTNER 2 study cohort (23). Relevant TR at baseline but not at the follow-up time points after TAVI was independently associated with 2-year mortality in our study.

In correspondence, Fan and co-workers could outline relevant baseline TR to be associated with increased all-cause mortality after TAVI (46). In contrast, a large French registry including 42.866 patients could not show that baseline TR independently predicted the mortality of patients who underwent TAVI (19).

Taken together, the extent of MR and TR at baseline as well as their dynamics after TAVI should be integrated into the individual risk assessment of elderly patients with AS. This is of particular importance since these parameters are mostly not included as covariates in established risk prediction models (47). Of note, despite not being an independent predictor in our analysis, relevant PVL at follow-up could be shown to be associated with reduced long-term survival rates. This goes in line with a variety of recent studies pointing out the prognostic role of relevant PVL after TAVI (48, 49).

Against the background of the availability of interventional treatment options of relevant MR and TR in terms of transcatheter edge-to-edge repair (TEER), the question arises of whether synchronous or contemporary treatment of MR or TR in patients with TAVI is of additional prognostic value. Since this question has not been answered concludingly until now, there are no clear recommendations for the clinical management of those patients in current guidelines (8, 50). To improve the situation, a randomized clinical trial addressing the effects of TEER on the mitral valve (clipping) in case of significant MR after TAVI has been initiated recently (MITAVI, NCT04009434).

In our current study, we could point out that also the follow-up time point is essential for decision-making with respect to TEER post-TAVI. Thus, although there was a significant improvement of relevant MR frequency already 6 to 8 weeks after TAVI with no further decrease after 6 months, the prognostic relevance of MR obviously demasked not until the 6-month follow-up. Of note, we here present a small single-center experience, but the results might sensitize for the phenomenon of reverse remodeling after TAVI as a complex interplay between different mechanisms finally determining patients’ prognosis (15, 29). This aspect should mandatorily be taken into account when interpreting future results of randomized trials in that important field.

The scenario of persisting relevant TR in patients after TAVI with or without significant MR (treated or not) is, in either case, complex and has not been addressed in clinical trials until now.

## Limitations

Although there are several clear strengths of our current study, e.g., its representative real-world character, the adherence to a standardized follow-up protocol including the assessment of dynamic changes of MR and TR, and the availability of complete mortality data at 2 years post-TAVI, there are also some limitations that need to be mentioned.

First, for outcome analysis after TAVI, the type of prosthesis implanted is of great impact because various outcome parameters significantly differ between valve types, in particular between balloon-expandable and self-expanding devices (51, 52). In our current study, the ratio between both prosthesis types not fully balanced, since 60.1% of the implanted prosthesis were balloon-expandable devices. This might be taken into account as a study limitation when interpreting the results. Moreover, the assessment of echocardiographic parameters including MR and TR before and after TAVI indeed was performed according to standardized in-center protocols, but no core laboratory was involved. Furthermore, the fact that the echocardiographic follow-up rate was not 100% at 6 to 8 weeks and 6 months has to be considered a limitation of the study, in particular with respect to potential immortal bias in mortality prediction and survival analyses.

## Conclusion

This real-life study demonstrated the prognostic relevance of repeated evaluation of MR and TR before and after TAVI. At least according to our current results, the prognostic impact of persisting relevant MR demasks not until 6 months after TAVI, whereas relevant TR predicts a worse prognosis before TAVI. Thus, choosing the right time point for treatment, e.g., by transcatheter edge-to-edge repair, is a remaining clinical challenge, which should be further addressed in randomized trials.

### Impact on daily practice

Because of prognostic importance, accurate and repeated evaluation of mitral and tricuspid regurgitation in patients with TAVI is strongly recommended.

## Data availability statement

The raw data supporting the conclusions of this article will be made available by the authors, without undue reservation.

## Ethics statement

The studies involving human participants were reviewed and approved by Ethics Committee of the University Hospital Jena. The patients/participants provided their written informed consent to participate in this study.

## Author contributions

LB, MF, SM-W, and PCS designed and performed the study. MD, TK, and KI contributed data to the study. LB and MF wrote the manuscript. SM-W and PCS revised the manuscript. All authors contributed to the article and approved the submitted version.

## Funding

This study was supported by funding from the Foundation “Else Kröner-Fresenius-Stiftung” within the Research Program “Else Kröner-Forschungskolleg AntiAge.” We acknowledge support by the German Research Foundation Projekt-Nr. 512648189 and the Open Access Publication Fund of the Thueringer Universitaets- und Landesbibliothek Jena.

## Conflict of interest

The authors declare that the research was conducted in the absence of any commercial or financial relationships that could be construed as a potential conflict of interest.

## Publisher’s note

All claims expressed in this article are solely those of the authors and do not necessarily represent those of their affiliated organizations, or those of the publisher, the editors and the reviewers. Any product that may be evaluated in this article, or claim that may be made by its manufacturer, is not guaranteed or endorsed by the publisher.
