# Supplementary material for: Prognostic relevance of mitral and tricuspid regurgitation after transcatheter aortic valve implantation: Impact of follow-up time point for decision-making
Source: Front Cardiovasc Med. 2023 Feb 16;10:990373. doi: 10.3389/fcvm.2023.990373 (PMC9977804; doi:10.3389/fcvm.2023.990373)
Supplement: Supplementary file 1 [file Table_1.docx]

**Supplementary Table 1:** *Results of Group Comparison regarding clinical, laboratory and echocardiographic parameters using the Mann-Whitney-U test to assess significant differences between long-term survivors and non-survivors (2-year mortality) at the different time-points (baseline, static and dynamic; 6 to 8 weeks and 6 months)*

| **Parameters** | **p value (Mann-Whitney-U test) between survivors and non-survivors after 2 years** |
| --- | --- |
| ***baseline_static (n=15)*** |  |
| Age | **0.035** |
| Female sex | **0.055** |
| AS subtype, other than HGAS | **0.002** |
| Afib | **<0.001** |
| Renal function (GFR) | **0.002** |
| Stage | **0.003** |
| CAD | **0.085** |
| PAD | **0.046** |
| LC | **0.061** |
| Height | 0.209 |
| Weight | 0.779 |
| Diabetes | 0.650 |
| COPD | 0.404 |
| PH | 0.220 |
| PM | 0.793 |
| ***baseline_dynamic (n=9)*** |  |
| SMWD | **<0.001** |
| PAPsys | **<0.001** |
| TR ≥ II | **<0.001** |
| BNP | **<0.001** |
| NYHA class | **0.003** |
| MR ≥ II | **0.049** |
| VAS | **0.066** |
| CFS | **<0.001** |
| LVEF | 0.489 |
| ***6 to 8 weeks follow-up (n=10)*** |  |
| CFS | **<0.001** |
| PAPsys | **0.002** |
| BNP | **<0.001** |
| NYHA class | **0.002** |
| SMWD | **0.004** |
| TR ≥ II | **0.081** |
| VAS | **0.005** |
| LVEF | 0.437 |
| MR ≥ II | 0.670 |
| AR ≥ II | 0.236 |
| ***6 months follow-up (n=10)*** |  |
| BNP | **0.001** |
| MR ≥ II | **0.047** |
| AR ≥ II | **0.058** |
| TR ≥ II | **0.033** |
| VAS | **0.048** |
| SMWD | 0.950 |
| NYHA class | 0.729 |
| LVEF | 0.342 |
| PAPsys | 0.378 |
| CFS | 0.230 |

*Abbreviations:* AS = aortic stenosis; HGAS = high gradient aortic stenosis; Afib = atrial fibrillation; GFR = glomerular filtration rate; stage = stages of extra-valvular cardiac damage; CAD = coronary artery disease; PAD = peripheral artery disease; LC = liver cirrhosis; COPD = chronic obstructive pulmonary disease; PH = pulmonary hypertension; PM = pace maker; SMWD = six minutes’ walk distance; PAPsys = systolic pulmonary artery pressure; TR = tricuspid regurgitation; BNP = brain natriuretic peptide; NYHA = New York Heart Association; MR = mitral regurgitation; VAS = visual analogue scale; CFS = clinical frailty scale; LVEF = left ventricular ejection fraction; AR = aortic regurgitation; p values < 0.1 are highlighted in bold letters.
